# Evidence for a Mixed Timing and Counting Strategy in Mice Performing a Mechner Counting Task

**DOI:** 10.3389/fnbeh.2019.00109

**Published:** 2019-06-25

**Authors:** Kenneth R. Light, Brian Cotten, Talia Malekan, Sophie Dewil, Matthew R. Bailey, Charles R. Gallistel, Peter D. Balsam

**Affiliations:** ^1^Department of Psychology, Barnard College of Columbia University, New York, NY, United States; ^2^Department of Psychology, Columbia University, New York, NY, United States; ^3^Department of Psychology, Rutgers University, Piscataway, NJ, United States

**Keywords:** counting, numerosity, timing, mice, operant conditioning

## Abstract

Numerosity, or the ability to understand and distinguish between discrete quantities, was first formalized for study in animals by Mechner (1958a). Rats had to press one lever (the counting lever) *n* times to arm food release from pressing a second lever (the reward lever). The only cue that *n* presses had been made to the counting lever was the animal’s representation of how many times it had pressed it. In the years that have passed since, many researchers have modified the task in meaningful ways to attempt to tease apart timing-based and count-based strategies. Strong evidence has amassed that the two are fundamentally different and separable skills but, to date, no study has effectively examined the differential contributions of the two strategies in Mechner’s original task. By examining performance mid-trial and correlating it with whole-trial performance, we were able to identify patterns of correlation consistent with counting and timing strategies. Due to the independent nature of these correlation patterns, this technique was uniquely able to provide evidence for strategies that combined both timing and counting components. The results show that most mice demonstrated this combined strategy. This provides direct evidence that mice can and do use numerosity to complete Mechner’s original task. A rational agent with fallible estimates of both counts made and time elapsed in making them should use both estimates when deciding when to switch to the second lever.

## Introduction

Numerosity is the ability of an organism to understand and distinguish between discrete quantities. Thus, counting to a certain number can be conceived of as a skill built on this ability because it is the ability to recognize when a pre-set number has been met or exceeded. To successfully count to a given number of responses, an organism must maintain an internal representation of the target number, keep track of the number of responses it has already made, and recognize when that number exceeds the target number.

Mechner (1958a,b) first tested the ability of rats to count on a task that required the rat to make a set number of responses or more (count requirement) on a counting lever before switching to a reward lever for a single response. These studies examined behavior under conditions where the count requirement was either 8 or 16. Interestingly, he found that under a variety of behavioral contingencies there was a robust pattern of behavior that appeared to indicate rats could effectively count out 8 or 16 responses before switching. Machado and Rodrigues (2007) replicated this study in pigeons using a more parametric approach, varying the count requirement from 4 to 32. They found that pecks increased linearly with count requirement, while the coefficient of variation (CV) remained fairly constant. In mice, Çavdaroğlu and Balcı (2016) demonstrated these same principles at count requirements of 10, 20, and 40.

Twenty-five years after Mechner’s publication, Meck and Church (1983) developed bisection procedures where rats were tasked with discriminating either the duration or number of sounds. One of two levers was assigned to the short duration (or small number) while the other was assigned to the long duration (or large number). They then played either intermediate times or intermediate counts to see which lever the rats responded on. Rats effectively discriminated 4:1 ratios in both timing and counting, and the point of subjective equality (where 50% of responses occurred on the “long” or “large quantity” lever) was close to the geometric mean of the ends of the distribution. Thus, rats demonstrated numerosity perception in this task at a level comparable to their ability to discriminate time. Fetterman and Killeen (2010) found similar results in pigeons performing on a three-lever switch task dependent on count.

The comparable levels of counting and timing ability make it hard to determine the rats’ true basis for the decision to switch levers. Timing and counting are nearly unavoidably correlated through rate. That is, a rat can both press a lever a given number of times or press at a certain rate for a certain duration of time and come to the appropriate number of lever presses before switching levers. Even when the stimulus is external, as in the bisection task, the presentation rate of stimuli confounds time and count. The two dimensions can be uncoupled by varying the duration of the cues to be counted and such experiments (Fernandes and Church, 1982; Meck and Church, 1983) showed that animals can use either time or count.

Attempts to tease apart counting and timing strategies in count production tasks have also been successfully done. Mechner and Guevrekian (1962) found that the speed of responses increased with deprivation on a fixed ratio schedule but not on a minimum interval schedule. Instead, on the interval schedule the break time between runs was decreased with increasing deprivation. Thus, time-based and response-based strategies were able to be isolated through deprivation. While it was not done in a counting task, it does demonstrate that they are separate, and separable, abilities in the rat.

Wilkie et al. (1979) attempted to dissociate timing and counting in a modified Mechner counting task in two pigeons. When they introduced a variable interval (but not a fixed interval) between the first and second pecks the animals made, it theoretically interfered with the animals’ ability to time this interval. Because performance was not perturbed by this variable interval (specifically in conditions 4 and 5) the authors concluded that timing was not necessary to complete the task.

The research cited above demonstrates that animals can both count and time but it does not indicate what the animals will do when both strategies are possible. To that end, Roberts and Mitchell (1994) demonstrated that pigeons faced with a stimulus that has both timing and number attributes can process both attributes simultaneously. Roberts et al. (2000) then demonstrated that these attributes can be brought under stimulus control with a discriminatory cue presented prior. Recently, Berkay et al. (2016) investigated animals’ strategies by modeling performance in a numerical switch task. This task, similar to the one employed by Fetterman and Killeen (2010), allowed the group to investigate the influence of when the animals’ chose to move from the “few” response lever to the “many” response lever. This switching behavior is indicative of the mouse discerning that the number of presses it made on the “few” lever was equal to or more than the number required for reward at that lever, causing it to begin responding on the “many” lever to earn a reward that trial. In this task, the group found evidence for behavior based on numerosity and only weak evidence that timing might also play some role in the performance. Intuitively one would expect animals to rely on the dimensions that they track with greatest precision. Both Fetterman (1993) and Çavdaroğlu and Balcı (2016) measured the variability of both timing and counting in the counting switch task and found that the variability of counting was less than that of timing. Further, Çavdaroğlu and Balcı (2016) showed that counting was more heavily weighted than timing in a regression analysis to predict the switch in response levers while Fetterman (1993) found individual differences in the use of timing and counting strategies.

**Figure 1:**
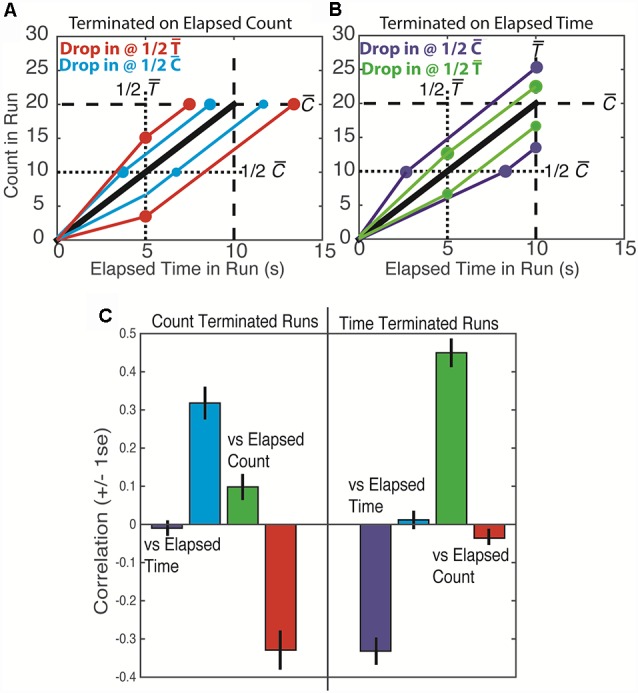
*Top*: schematic plots of press count vs. elapsed run time. The black line is the average across many trials. The colored lines portray individual runs that are found to be slower or faster than the average at 1/2 the mean terminal time [1/2 $\bar{T}$ and red **(A)** and green **(B)** lines/dots] or at 1/2 the mean terminal count [1/2 $\bar{C}$ and blue **(A)** and purple **(B)** lines/dots]. Lines equal in slope to the average slope extend from these drop-in points to the mean terminal time $\bar{T}$ and mean terminal count $\bar{C}$. Bottom: the correlations obtained from simulated data. The colors of the lines/dots in the top panel denote the predicted correlations, as portrayed in the bottom panel by bars of the same color. Thus, for example, a low count at 1/2 $\bar{T}$ predicts a low terminal count if the run is time terminated (panel **B**, lower green line/dots) and a long terminal time if the run is count terminated (panel **A**, lower red line/dots) The first prediction is the positive correlation—low with low and high with high—that was in fact observed in our simulation (green bar on the right side of the lower panel). The second prediction is the negative correlation—low with long and high with short—that was in fact observed in our simulation (red bar on the left side of the lower plot).

The present study sought to discern whether counting and/or timing guided behavior in the original Mechner counting task. In this task, the number of presses made on the counting lever before switching to the reward lever (which will be called terminal count) and the time it takes to make those presses (called the terminal time) are highly correlated. A way of discerning whether counting or timing is the basis of the decision to terminate presses on the counting lever arises from the fact that the rate of pressing varies from trial to trial because pressing is often interrupted by pauses of varying duration at varying points in the sequence. Because of these irregularities, when one “drops in” analytically (*post hoc*) on runs at a fixed time equal to half the average terminal time, the counts at that fixed time vary. The correlations between the varying elapsed counts observed at a fixed drop-in time and the terminal times and terminal counts depend on whether the animal counts its presses or times the elapsing interval(s) [Figure 1, red **(A)** and green **(B)** lines/dots]. Similarly, when one drops in at a fixed count equal to half the average terminal count, the times elapsed at that count vary [Figure 1, blue **(A)** and purple **(B)** lines/dots]. The correlations between the varying times elapsed at a fixed drop-in count and the terminal counts and terminal times depend on whether count or elapsed time or both is the basis of the animal’s behavior.

We estimate the extent to which behavior was based on counting or timing or both by computing the four just-described drop-in correlations: terminal count and terminal time vs. drop-in count and terminal count and terminal time vs. drop-in time. When the drop-in is at a fixed time, t, and a counting strategy is being used, the following quantitative relations are relevant:

*C* = *c*_t_ + *c*_r_, where $c_{r}=\bar{C}-c_{t}$. The terminal count is the sum of the count at drop-in time t, (*c*_t_) and the count remaining to reach the target (*c*_r_). If *the count* is the sole basis for terminating a run of presses on the counting lever, then $\bar{C}$ is determined entirely by the target count. In that case, the counts remaining are the average terminal count minus the counts at drop-in.
*T* = *t* + *t*_r_ the terminal time on a temporal drop-in trial is the fixed drop-in time, *t*, plus *t*_r_ the time remaining.
*t*_r_ = *c*_r_/λ the time remaining is the count remaining divided by the average rate of pressing; the latter is a constant, so the smaller *c*_r_ is, the shorter *t*_r_ is.

By substitution: $T=t+c_{r}/\lambda =(\bar{C}-c_{t})/\lambda$. Because t, $\bar{C}$ and λ are constants, a bigger than usual *c*_t_ predicts a smaller than usual *c*_r_, which predicts a shorter than usual *t*_r_, which in turn predicts a shorter than usual *T*. Thus, *T* (terminal time) should be negatively correlated with *c*_t_ (Figure 1C, red bar on the left side). On the other hand, *C* (terminal count) should not be correlated with *c*_t_ because a shorter than usual *C*_r_ offsets the effect on *C* of a higher than usual *c*_t_ (Figure 1C, green bar on the left side).

When the analytic drop-in is at a fixed count, c, rather than at a fixed time and a counting strategy is being used, then total time, *T* = *t*_c_ + *t*_r_ should be longer than usual when *t*_c_ is longer than usual, because the average count remaining to the target, hence the expected time remaining, *t*_r_, should be a constant. Thus, *T* should be positively correlated with *t*_c_ (Figure 1C, blue bar on the left side), but *C* should not be correlated with *t*_c_ (Figure 1C, purple bar on the left side).

A parallel analysis of what is expected when the termination of a run of presses on the counting lever is based solely on timing an interval (either the elapsed *trial* time or the elapsed *run* time) yields the pattern of correlations shown in Figure 1C on the right side.

As a check on the soundness of our analytic derivation of these correlation patterns, we did two simulations. In the first, we chose a terminal count, *n*, for each simulated run at random from among the distribution of those counts for a given subject. We then randomly chose inter-response intervals from the distribution of inter-response intervals for that subject. We cumsummed them to obtain response times for the *n* responses. In this simulation, the decision to end a sequence only depends on count as it would for a pure counting strategy. In the second simulation, we chose terminal run times at random from among the distribution of those times for a given subject. For each terminal run time, we then chose a long sequence of inter-response intervals at random from the distribution of those intervals for a given subject. We cumsummed these intervals and truncated the sequence at the end of the inter-response time that exceeded the target interval. Thus, the decision to end a sequence in this second simulation depended only on elapsed run time, as it would for a pure timing strategy. We then computed all of the correlations as we did with the real data. The drop-in correlation patterns on the simulated data were those predicted by our derivations (Figure 1).

Importantly, this correlational analysis can reveal strategies based on mixtures of count and elapsed times. Such strategies will yield mixtures of the “pure” patterns shown in the top panels of Figures 4, 5. We use this correlation analysis in considering five possible strategies that subjects might use to decide when to terminate pressing the counting lever and switch to the reward lever: (i) a strategy based purely on the press count; (ii) a strategy based purely on the elapsed *trial* time; (iii) a strategy based purely on the elapsed *run* time (time from the first response); (iv) a strategy based on both the press count and the elapsed trial time; and (v) a strategy based on the press count and the elapsed *run* time.

**Figure 2:**
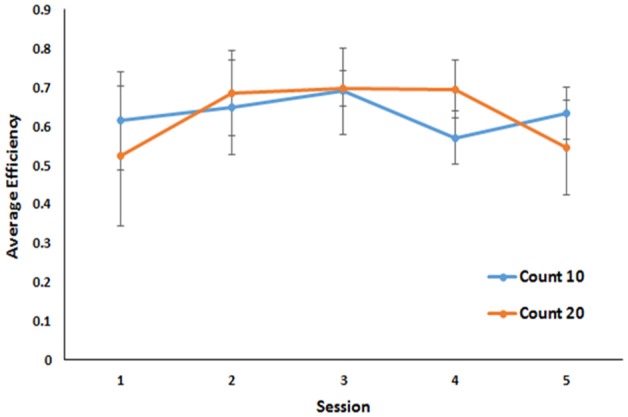
Performance across the last five sessions of a Mechner counting task. Average efficiency is plotted as a function of session for both 10 and 20 count requirements. Error bars indicate standard errors of the mean.

**Figure 3:**
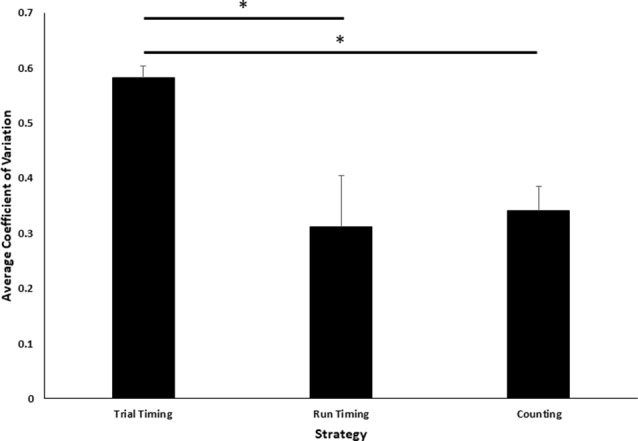
Comparison of coefficient of variation (CV) across task strategies. Average CV of terminal values across the last five sessions of training is plotted as a function of three possible strategies. Error bars indicate standard errors of the mean. Asterisks indicate statistically significant LSD *post hoc* comparisons (*p* < 0.05).

**Figure 4:**
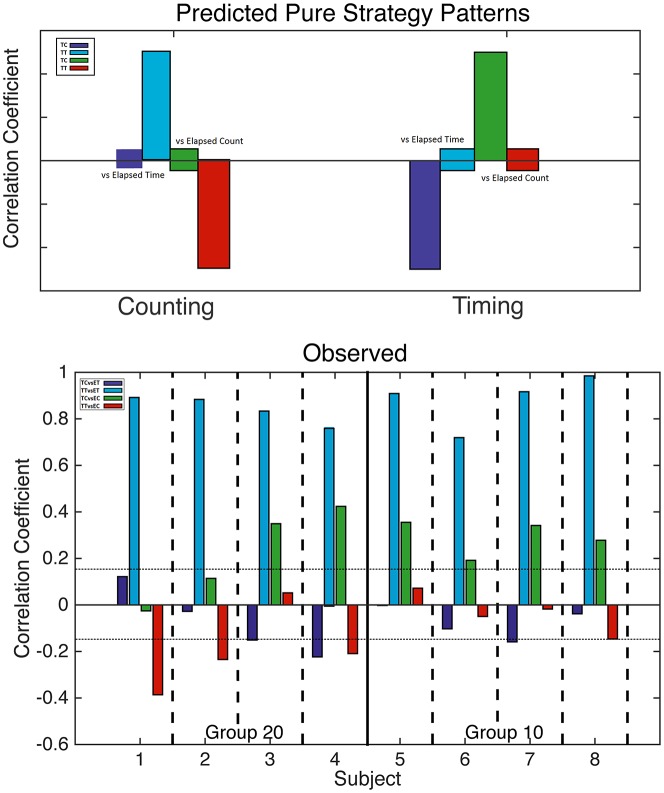
Analysis of counting and trial timing strategies. Top: predicted correlations based on counting (left) and trial timing (right) strategies. When one drops in at a fixed count, only elapsed *trial* time varies; likewise, when one drops in at a fixed *trial* time, only elapsed count varies. Bottom: computed correlation coefficients for all four mice with a 20-count requirement (left) and all four mice with a 10-count requirement (right). The horizontal dotted lines mark alpha = 0.01; bars that cross these lines indicate correlations significant at beyond the 0.01 level.

**Figure 5:**
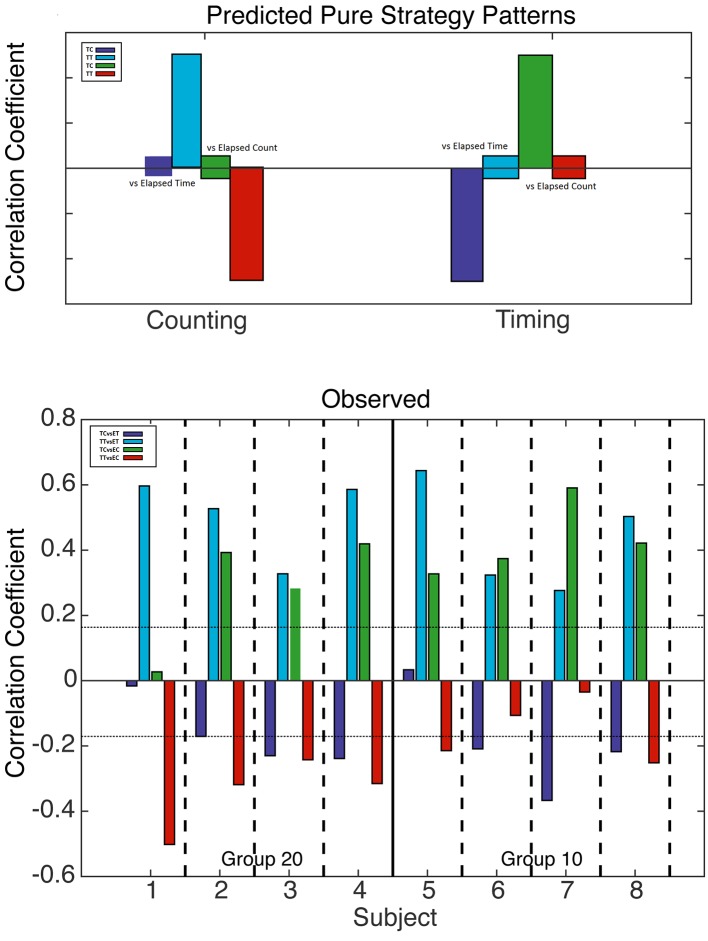
Analysis of counting and run-time timing strategies. Top: predicted correlations based on counting (left) and run-time timing (right) strategies. When one drops in at a fixed count, only elapsed *run* time varies; likewise, when one drops in at a fixed *run* time, only elapsed count varies. Bottom: computed correlation coefficients for all four mice with a 20 count requirement (left) and all four mice with a 10 count requirement (right). The horizontal dotted lines mark alpha = 0.01; bars that cross these lines indicate correlations significant at beyond the 0.01 level.

## Materials and Methods

### Subjects

Eight adult male C57/BL6 mice (Jackson Labs, Bar Harbor, ME, USA) were used in this experiment. All mice were kept in standard laboratory cages housed four per cage in a temperature and humidity controlled vivarium on a 12 h light/12 h dark cycle with a light onset of 7 am. *Ad libidum* access to water was maintained at all times while the animals were within their home cages. Feeding was restricted to maintain 85% of free-feeding body weight.

### Apparatus

Mouse modular operant chambers (MED Associates, Fairfax, VT, USA) were used for all behavioral assessments in this study. The chambers were equipped with grid flooring, a house light, two retractable levers that flanked a feeding trough, and a dipper arm able to deliver 0.01 cc of evaporated milk.

### Procedure

Mice were acclimated to the vivarium for 1 week prior to experimental testing. During this time, they were also acclimated to handling through taking their body weight daily. This baseline weight was then used to restrict food access as outlined above.

The first phase of training was dipper and lever press training, which occurred over two consecutive daily sessions. During both sessions, animals were rewarded with a drop of milk if they pressed the left lever. In session 1, the lever was extended and milk was delivered automatically after 30 s. A variable ITI with an average of 5 s then started before the lever extended again, starting the next trial. This continued for 20 trials. The second session was similar to the first, but the lever retracted after 20 s and the procedure continued for 30 trials.

Next, mice were trained on a forced response chain that increased the Fixed Ratio (FR) requirement for the first lever (“count”) over days. The FR requirement for the second lever (“reward lever”) was always 1. To achieve a forced chain, the count lever was extended until the FR requirement was met, at which point it retracted, the reward lever was extended, and a single press on the reward lever always resulted in a drop of milk. After the reward, the reward lever retracted and a variable ITI with an average of 15 s occurred before the count lever was again extended, starting the next trial. Subjects continued in this manner until 40 rewards were earned or 60 min elapsed. Subjects progressed through FR requirements of 1, 2, 3, 4, and 5 at individual paces before moving on to the next phase.

In the final phase of training, the response chain was no longer forced. Instead, both levers were extended at the start of a trial and both were retracted at the end. The trial only resulted in the presentation of a drop of milk if the mouse made at least the required number of responses on the count lever before switching to the reward lever for one press. Extra presses on the count lever were never penalized. Like the previous phase, this procedure continued until 40 rewards were earned or 60 min elapsed. All mice proceeded through requirements of 5, 7, and 10 presses on the count lever at individual paces. A subset of mice (*n* = 4) were then gradually moved to a requirement of 20 presses. All mice were then trained on their final requirement (10 or 20) for a minimum of 15 sessions.

## Results

Because this task required animals to perform until they obtained 40 rewards, the number of trials taken to reach those rewards is indicative of their ability to perform the task as well as the efficiency of the strategy they employed to perform it. However, not all subjects earned all 40 rewards every session (subject 2 earned no rewards in session 1 and only 22 in session 5; subject 3 earned 38 rewards in session 5) before the 60-min time out. Therefore, a better measure of efficiency is the number of rewards earned divided by the number of trials taken to earn them. By that metric, in the final five sessions of training, all animals achieved a stable level of efficiency. No differences in overall efficiency were observed for mice under the 10 press requirement and mice under the 20 press requirement (M = 0.64 in both cases BF 1.9:1 in favor of the null against a bidirectional effect size of ±0.25). No group by trial interaction was observed (Figure 2).

Intuitively, one might expect that when there are two evolving and correlated cues that may determine a decision, the cue on which the decision is based will have less variable terminal values, hence a smaller CV. Consequently, we compared the CVs of the three terminal quantities (trial time, run time and press count). The CVs differ significantly, *F*_(2,21)_ = 5.08, *p* < 0.05 (Figure 3). Least-significant-difference *post hoc* analyses revealed that the average trial timing CV was significantly larger than both the run-time (*p* < 0.05) and the count (*p* < 0.01) CVs, but the latter two did not differ (*p* = 0.75).

The larger trial timing CV does not definitively exclude the possibility of a trial-timing strategy contributing to the animals’ decision to switch to the reward lever. To gather further relevant evidence, the four correlations described in the introduction were computed for each animal for the terminal trial times.

The correlations predicted by the two “pure” strategies are shown in the upper panel of Figure 4, while the correlations actually obtained are shown in the bottom panel. In the bottom panel, we see that the blue correlations were strongly positive in every subject, as is predicted by a counting strategy. A pure counting strategy also predicts that the red correlations should be strongly negative. This is true for only half the subjects; in the other half, the red correlations are non-significant. A pure counting strategy predicts non-significant green correlations, but these are significantly positive in six subjects, as predicted by a timing strategy. Thus, the results of a correlational analysis of elapsed *trial* times and counts imply that all the subjects terminated pressing on the left lever on the basis of the number of presses at least some of the time, but that the time elapsed played a role in their decision on at least some runs.

We conducted the same analysis—with two different drop-ins and the four correlations—with elapsed *run* times rather than elapsed *trial* times. In this analysis, the fixed drop-in time was half the average terminal run time, that is, half the interval from the first to the last press on the count lever. If the decision to terminate pressing is based on the time elapsed since the first press rather than on the time elapsed since the start of the trial, the time correlations will get stronger; hence the overall pattern of correlations might become more mixed.

In shifting from Figure 4 to Figure 5, one sees that the evidence for a timing strategy increases in most subjects. Indeed, in Subject 7, the correlations expected from timing are significant, whereas the correlations expected from counting are non-significant. The pattern in Subject 7 contrasts strikingly with the pattern in Subject 1, whose pattern is exactly what is expected given a pure counting strategy. The other six subjects show clear evidence of dependence on both counting and timing.

Next, we attempted to estimate the contributions of counting-based and timing-based decisions in individual animals from the run time analyses. To this end, we subtracted the correlation predicted to be negative for each contributor from the correlation predicted to be positive. That is, for counting we subtracted the correlation between terminal time and current count from the correlation between elapsed time and terminal time. For timing, we subtracted the correlation between terminal time and elapsed time from the correlation between current count and elapsed time. We were then able to calculate the individual animals’ bias towards one strategy or the other by subtracting our timing measure from our counting measure. Thus, mice with biases towards count as the more important basis of their decision to switch levers had a positive value and mice with biases towards time as the more important basis of their decision to switch levers had a negative value.

As a group, mice had an average bias of 0.046, indicating that generally mice show no clear bias towards one estimate or the other. However, the group mean is misleading because individual differences in this measure are very large. The range of values was from −0.80 to +0.92 because mouse seven relied on a pure timing strategy while mouse 1 relied on a pure counting strategy. Most, however, showed evidence for both timing and counting with a mild bias toward one or the other.

**Figure 6:**
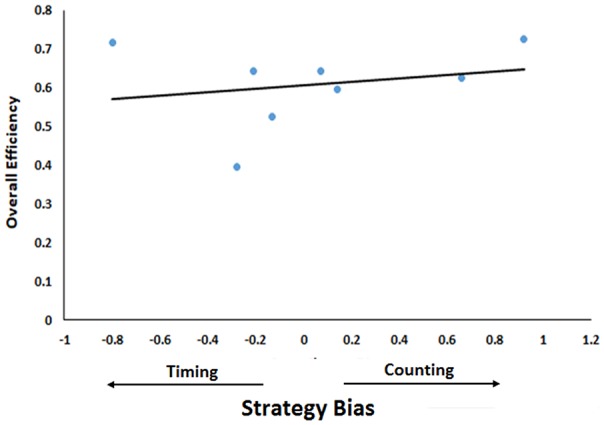
Relationship between efficiency and bias towards one strategy. Strategy Bias (difference between contribution of timing and counting) is plotted as a function of Overall Efficiency (number of rewards earned/number of trials for all five sessions). Positive values of contributor bias indicate counting bias while negative values indicate timing bias. The trend line indicates the regression line.

Finally, we correlated mice’s efficiency at completing the task (see above) with this measure of bias towards one strategy or the other. However, due to the small sample size here, we cannot determine statistical significance, we can only examine coefficients as descriptive of our sample. There was a small positive correlation between the bias score and efficiency (*r* = 0.23) suggesting that biases towards counting are more effective than biases towards timing (Figure 6). However, it is noteworthy here that the mouse that was heavily biased towards timing and the mouse that was heavily biased towards counting were the two most efficient mice in the sample. Further, if one measures the correlation among only those that had a substantial count-basis for their decision (biases equal to −0.3 or larger), the correlation rises to 0.69. The same cannot be said when we examine only those with substantial timing-based biases (biases equal to 0.3 or smaller). The correlation then reverses to −0.26, indicating that when added to a counting strategy, timing is detrimental. Generally, then, choosing one variable to base a decision on is best and mixing one’s strategy comes at the sacrifice of efficiency.

## Discussion

In the present experiment, a Mechner counting task was evaluated in terms of the animals’ abilities to complete the task at 10 and 20 press requirements and the strategies employed to accomplish the task. All mice presented here were able to complete a 10 press requirement and the subset tasked with completing a 20 press requirement did so effectively. Further, no differences in efficiency in completing the task were observed for the different press requirements.

Three variables were considered as possible contributors to the decision to switch levers in this analysis: one count-based and two timing-based contributors. The count-based contributor was the one conceived of in the initial design of the task. That is, mice could track, or count, the number of times they pressed the counting lever and switch to the reward lever once some target number is reached. The first timing-based contributor considered here assumes tracking the time elapsed in a trial. On this assumption, mice track the time from when the levers are inserted into the chamber (the start of the trial) and press until some target trial time is reached. The second timing-based strategy here is a press-run timing strategy. In this case, mice track the time elapsed since their first press on the counting lever until a target elapsed run time is reached.

First, and most importantly, all but one of our subjects based their decision at least in part on counting—in support of the original intention of the task (Mechner, 1958a,b), as well as decades of previous work manipulating the task (Mechner and Guevrekian, 1962; Wilkie et al., 1979; Machado and Rodrigues, 2007; Fetterman and Killeen, 2010) and analyzing data from similar tasks (Berkay et al., 2016; Çavdaroğlu and Balcı, 2016). Most mice, regardless of count requirement and which time (trial or run) the count strategy was compared to, demonstrated a count-based contribution to the decision to switch levers.

The correlational analysis of each animal’s efficiency across the final five sessions of training indicated that a strategy based on elapsed *trial* time is less predictive of accuracy than one based on either run time or counting as might be predicted from the greater relative variability of trial times compared to the other measures. In the correlational efficiency analysis, comparison of the run time strategy alongside of a count-based strategy revealed that most mice used a combination of the two. However, in terms of efficiency, the data seem to suggest that while choosing a nearly pure timing strategy was very effective, when using a more mixed strategy the greater contribution counting made to the decision to switch levers, the more efficiently mice performed the task.

It is the finding that a combined strategy seems to be the most prevalent that is simultaneously the most revealing of the manner in which animals approach this task and others like it as well as the largest strength of this type of analysis. To understand why this is the case, one must understand first that, because count alone determines the outcome of this task, the optimal strategy here would be to count precisely. However, mice cannot count with sufficient precision to produce error-free performance. Therefore, adding a secondary strategy, such as timing a response-run, could lead to rewarded trials that would otherwise have been error trials due to erroneous counts. The evidence here seems to indicate that adding a second strategy may sacrifice efficiency. Presumably, mice would otherwise not be able to complete the task so, that sacrifice is often worth it. Interestingly, Roberts et al. (2000) presented evidence that, at least in pigeons, this compensatory strategy is only present when the animal is counting, but not timing. Due to the counting nature of the task, however, we are unable to make that distinction here.

Finally, the present results have implications for the way complex tasks tend to be analyzed, both in animal models and in humans. By parsing out different strategic contributions for individuals, one can then use a single task to measure multiple abilities. It would then be possible to manipulate one trait and see how that affects the individual contributors to the task as well as how the combination of those strategies might be altered. To use the present line of research as an example, one could alter animals’ abilities to time and see how that affects their abilities to time and count on the Mechner task as well as whether a lack of timing ability makes them rely more heavily on a counting strategy. In this manner a much more holistic view of mouse performance becomes possible.

## Ethics Statement

This study was carried out in accordance with the recommendations of National Institutes of Health (NIH) Guide for Animal Care and Use and the recommendations of the IACUC of Columbia University and New York State Psychiatric Institute. The protocol was approved by the IACUC of Columbia University and New York State Psychiatric Institute.

## Author Contributions

KL contributed to the design of the study, analysis of the data, writing of the manuscript. BC, TM, and SD contributed to the design and execution of the study. MB contributed to the design and analysis of the study. CG contributed to the analysis of the study and editing of the manuscript. PB contributed to the design of the study, analysis of the data, and editing of the manuscript. All authors contributed intellectually to this study.

## Conflict of Interest Statement

The authors declare that the research was conducted in the absence of any commercial or financial relationships that could be construed as a potential conflict of interest.
